# Endothelial Arginine Resynthesis Contributes to the Maintenance of Vasomotor Function in Male Diabetic Mice

**DOI:** 10.1371/journal.pone.0102264

**Published:** 2014-07-17

**Authors:** Ramesh Chennupati, Merlijn J. P. M. T. Meens, Vincent Marion, Ben J. Janssen, Wouter H. Lamers, Jo G. R. De Mey, S. Eleonore Köhler

**Affiliations:** 1 Department of Anatomy & Embryology, Maastricht University, Maastricht, the Netherlands; 2 Department of Pharmacology, Maastricht University, Maastricht, the Netherlands; 3 Cardiovascular Research Institute Maastricht (CARIM), Maastricht University, Maastricht, the Netherlands; 4 Nutrition, Metabolism and Toxicology (NUTRIM) of Maastricht University, Maastricht, the Netherlands; 5 Department of Pathology and Immunology, University of Geneva, Geneva, Switzerland; 6 Department of Cardiovascular and Renal Research, Institute of Molecular Medicine, University of Southern Denmark, Odense, Denmark; 7 Department of Cardiovascular and Thoracic Surgery, Odense University Hospital, Odense, Denmark; University of Pittsburgh School of Medicine, United States of America

## Abstract

**Aim:**

Argininosuccinate synthetase (ASS) is essential for recycling L-citrulline, the by-product of NO synthase (NOS), to the NOS substrate L-arginine. Here, we assessed whether disturbed arginine resynthesis modulates endothelium-dependent vasodilatation in normal and diabetic male mice.

**Methods and Results:**

Endothelium-selective *Ass*-deficient mice (*Ass^fl/fl^/Tie2Cre^tg/−^ = *Ass-KO^Tie2^) were generated by crossing *Ass^fl/fl^* mice ( = control) with *Tie2Cre* mice. Gene ablation in endothelial cells was confirmed by immunohistochemistry. Blood pressure (MAP) was recorded in 34-week-old male mice. Vasomotor responses were studied in isolated saphenous arteries of 12- and 34-week-old Ass-KO^Tie2^ and control animals. At the age of 10 weeks, diabetes was induced in control and Ass-KO^Tie2^ mice by streptozotocin injections. Vasomotor responses of diabetic animals were studied 10 weeks later. MAP was similar in control and Ass-KO^Tie2^ mice. Depletion of circulating L-arginine by arginase 1 infusion or inhibition of NOS activity with L-NAME resulted in an increased MAP (10 and 30 mmHg, respectively) in control and Ass-KO^Tie2^ mice. Optimal arterial diameter, contractile responses to phenylephrine, and relaxing responses to acetylcholine and sodium nitroprusside were similar in healthy control and Ass-KO^Tie2^ mice. However, in diabetic Ass-KO^Tie2^ mice, relaxation responses to acetylcholine and endothelium-derived NO (EDNO) were significantly reduced when compared to diabetic control mice.

**Conclusions:**

Absence of endothelial citrulline recycling to arginine did not affect blood pressure and systemic arterial vasomotor responses in healthy mice. EDNO-mediated vasodilatation was significantly more impaired in diabetic Ass-KO^Tie2^ than in control mice demonstrating that endothelial arginine recycling becomes a limiting endothelial function in diabetes.

## Introduction

The endothelium regulates vasomotor tone by releasing several relaxing (endothelium-derived relaxing factors, EDRF) and contractile factors (EDCF). The major relaxing factors are nitric oxide (NO), prostacyclin (PGI_2_) and endothelium-dependent hyperpolarization (EDH). NO is not only an important vasodilator, but also inhibits atherogenic processes, such as smooth muscle-cell proliferation, platelet adhesion and aggregation and oxidation of low-density lipoproteins (LDL) [1]–[4]. Several studies demonstrated an impaired production of endothelial NO in patients with hypertension, heart failure, hypercholesteremia, atherosclerosis, and diabetes [5]–[9]. Nitric-oxide synthases (NOS) produce NO from the substrate arginine. Reported intracellular concentrations of arginine vary between 300 [10] and 800 µM [11], which is much higher than the K_m_ (3 µM) for endothelial NOS (NOS3). Despite this high intracellular arginine concentration, improved NO production [11] or improved endothelial function of small coronary vessels [12] have been reported after arginine supplementation. This phenomenon, which is known as the arginine paradox [13], [14], shows that the intracellular arginine concentration can become limiting under some conditions. Intracellular availability of arginine depends on transport, recycling, metabolism and catabolism [15].

Arginine can be resynthesized from citrulline, the by-product of NO production, via argininosuccinate synthetase (ASS) and argininosuccinate lyase (ASL). Both enzymes are expressed in many cell types [16]. Arginine is catabolized by arginases to ornithine and urea. The two isoforms, arginase 1 (cytoplasmic, also known as liver-type) and arginase 2 (mitochondrial, also known as kidney-type) are both reported to be expressed in endothelial cells [17], [18]. An increased activity of both arginase 1 and arginase 2 was demonstrated in diabetes and aging [19], [20], two conditions, which are associated with decreased NO production.

Although intracellular arginine sources for NOS3 are controversial, previous *in-vitro* studies have shown that arginine recycling is important for NO production [21]. It has, however, not yet been demonstrated whether this system is also relevant in endothelial cells *in vivo*. We hypothesize that deficient arginine resynthesis from citrulline in the endothelium predisposes to endothelial dysfunction (ED), which will be aggravated in diabetes. We tested this hypothesis in mice with a genetically impaired capacity to recycle arginine in their endothelium and investigated their saphenous arteries. We have previously shown that upon aging endothelium-dependent relaxing responses to acetylcholine become predominantly mediated by endothelium-derived NO in these muscular resistance arteries [22].

## Materials and Methods

### Animals

All procedures were performed in accordance with the guidelines of the Committee for Animal Care and Use of Maastricht University and have been approved by this Committee. Approval numbers for the protocols used in this study were: DEC 2008-182 and DEC 2012-027. Animals were killed by CO2/O2 inhalation to isolate blood vessels for vascular reactivity measurements.

For invasive hemodynamics, indwelling catheters were placed under isoflurane anesthesia. Analgesia was obtained by perioperative subcutaneous injections of buprenorphine (0.03 mg/kg). Two days after introduction of the catheters, blood pressure was measured in conscious animals. After the experiments, animals received 250 mg/kg pentobarbital through the catheter for euthanasia.

Endothelial *Ass-*deficient (*Ass^fl/fl^/Tie2Cre^tg/−^*) mice were generated by crossing animals carrying the floxed allele *Ass^fl/fl^* [23] with *Tie2Cre* mice. The endothelial knockout animals will be designated as Ass-KO^Tie2^, and the *Ass^fl/fl^* mice as controls. We have previously shown that *Ass^fl/fl^* mice are indistinguishable from their wild type littermates [23]. 12- and 34-week-old male and female mice were used for the experiments. Animals were housed in standard cages (constant room temperature and humidity, 12 hr light/dark cycles) and had free access to standard chow (pellets) and tap water. Diabetes was induced at the age of 10 weeks by intraperitoneal (IP) injections of streptozotocin (STZ, 50 mg/kg) for 5 consecutive days (AMDCC protocols; https://www.diacomp.org). Fasting blood glucose was measured after 1, 4, and 10 weeks following STZ injections, and male mice with stable blood glucose levels of ≥20 mmol/L were used for the experiments (mean ± SEM: 22±0.7 mmol/L, n = 8). Female mice were excluded from these experiments due to low fasting blood glucose levels (mean ± SEM: 7.7±0.3 mmol/L, n = 11; Table S1) 10 weeks after the streptozotocin treatment.

### Solutions and drugs

Krebs Ringer bicarbonate-buffered salt solution (KRB) contained (in mM): 118.5 NaCl, 4.7 KCl, 2.5 CaCl_2_, 1.2 MgSO_4_, 1.2 KH_2_PO_4_, 25.0 NaHCO_3_ and 5.5 glucose. The KRB solution was continuously aerated with 95% O_2_/5% CO_2_ and maintained at 37°C. Indomethacin (INDO; Sigma Aldrich, Zwijndrecht, NL) was dissolved in ethanol. Acetylcholine (ACh), noradrenaline (NA), phenylephrine (PHE), N^ω^-nitro-arginine methyl ester (L-NAME) and sodium nitroprusside (SNP; all Sigma Aldrich) were dissolved in KRB solution. High K^+^-KRB solution was prepared by replacing NaCl with KCl. Buffers containing intermediate K^+^ concentrations were prepared by mixing the appropriate volumes of KRB and K^+^-KRB.

### Immunohistochemistry

Saphenous arteries were fixed in 4% phosphate-buffered formalin at room temperature (RT) for 4 hrs and embedded in paraffin. Sections (4 µm thick) were rehydrated and boiled in sodium citrate buffer (10 mM, pH 6.0) for 15 min for epitope retrieval. Subsequently, sections were incubated overnight at 4°C in a humidified chamber with rabbit antibodies directed against ASS (1∶10,000 in normal goat serum (NGS); AMC, Amsterdam [24]). Excess antibody was washed off with PBS before sections were incubated with horseradish peroxidase-coupled rabbit antibodies against sheep IgG (1∶400 in NGS, DAKO, Glostrup, DK). The localization of HRP was visualized with 3, 3,-diaminobenzidine (Sigma Aldrich). The presence of ASS was visualized with an Axioscope (Carl Zeiss, Jena, Germany) and a standard charge-coupled digital camera (model DFC 280; Leica, Wetzlar, Germany).

### Plasma amino-acid analysis

For the determination of plasma amino acids, 50 µL of plasma was added to 4 mg sulfosalicylic acid, vortexed, snap-frozen in liquid nitrogen and stored at −80°C until use. The acid plasma supernatant was used for amino-acid analysis on a gradient reversed-phase HPLC system as described [25]. Prior to separation on a BDS Hypersil C18 column (Thermo Scientific, Breda, The Netherlands), the amino acids were labeled with o-phtalaldehyde (Pierce)/3-mercaptopropionic acid (Sigma). The amino acids were eluted with an acetonitrile gradient (0.7 mL/min) and fluorescence detected at 450 nm (excitation: 340 nm).

### *In vivo* hemodynamics

We determined hemodynamic parameters in conscious, unrestrained control and Ass-KO^Tie2^ mice. Heparinized indwelling polyethylene catheters were introduced under isoflurane anesthesia into the femoral artery and jugular vein, two days before the experiments. Analgesia was obtained by perioperative subcutaneous injections of buprenorphine (0.03 mg/kg). On the day of the experiment, the arterial line was connected to a pressure transducer (Micro Switch 150 PC) and the signal was sampled at 2.5 kHz. MAP and heart rate (HR) were derived from this signal using the IDEEQ data acquisition system (Instrument Services, Maastricht University, NL). The venous line was extended outside the cage and filled with 0.9% NaCl solution. Hemodynamic parameters were allowed to stabilize before pharmacological interventions. The following compounds were tested: bovine arginase 1 (200 U (Cell Sciences, Canton, MA, USA), dissolved in HEPES buffer (pH 7.4) and administered as an intravenous (IV) bolus), L-NAME (10 mg/kg, dissolved in 0.9% saline, IV), and ACh (7 µg/kg.min, dissolved in 0.9% NaCl, IV infusion). After the experiments, animals were euthanized with 250 mg/kg pentobarbital applied through the catheter. We were not able to cannulate the femoral arteries of diabetic mice because of the poor general condition of these animals and the reduction of vessel size.

### *In vitro* studies

#### Tissue preparation

Saphenous arteries, muscular resistance arteries with a diameter of ∼250 µm that are well suited to assess vasomotor responses *ex vivo* [22], [26], were used to study endothelium-dependent vasodilatation. Animals were euthanized by CO_2_/O_2_ inhalation. The arteries were dissected free from surrounding fat and connective tissue, and were mounted in a wire myograph (Danish MyoTechnology, Aarhus, DK). Arterial segments (2 mm long) were distended to the diameter at which maximal contractile responses to 10 µM NA (noradrenaline) were obtained [22], [27]. Optimal diameters (D_opt_) and maximal contractile responses to NA for male mice are summarized in Table S2.

#### Contribution of NO, EDH and cyclooxygenase products to endothelium-dependent relaxation

A concentration-response curve (CRC) for PHE (0.01–10 µM) was recorded. During the contraction induced by 10 µM PHE, a CRC for ACh (0.01–10 µM) was generated. Thirty min later, arteries were contracted using K^+^ (40 mM) and again a CRC for ACh (0.01–10 µM) was recorded. These experiments were repeated in the presence of the cyclooxygenase inhibitor indomethacin (INDO, 10 µM) and in the presence of both INDO and the NOS inhibitor L-NAME (100 µM).

#### Sensitivity of vascular smooth muscle to NO

Arteries were contracted with PHE (10 µM) in the presence of INDO (10 µM) and L-NAME (100 µM), and the relaxing effects of the NO donor SNP (0.01–10 µM) were recorded.

### Statistical analysis

All CRCs for contractile stimuli were expressed as percentage of the maximal response to 10 µM NA prior to the administration of any pharmacological inhibitor. Relaxing responses were expressed as percentage of the level of pre-contraction. Individual CRCs were fitted to a sigmoid regression curve (Graphpad Prism 5.0). Sensitivity (pEC_50_) and maximal effect (E_max_) are shown as means ± SEM. Unpaired Student’s t-tests were performed to determine the significance of differences in pEC_50_ and E_max_.

## Results

### Ablation of *Ass* gene from endothelial cells

**Figure 1:**
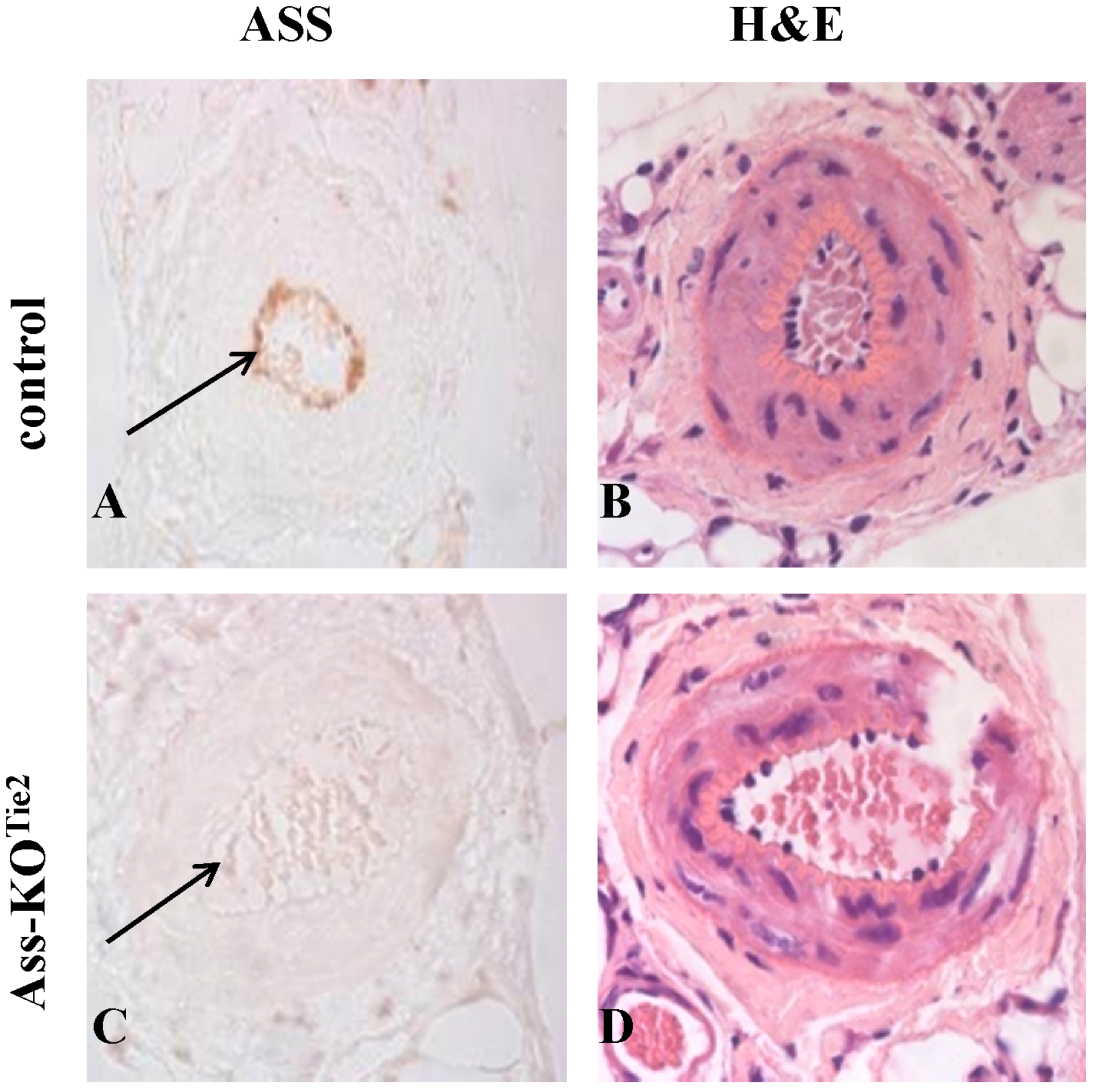
Expression of ASS protein in saphenous arteries of male control (panel A) and Ass-KO^Tie2^ (panel C) mice. The arrow indicates endothelial expression in control animals (panel A) and absence of endothelial expression in Ass-KO^Tie2^ mice (panel C). Panels B and D represent the corresponding H&E staining of a serial section to demonstrate the presence of intact endothelium.

To verify that there was indeed no ASS expression in the endothelium of Ass-KO^Tie2^ mice, paraffin-embedded saphenous arteries were sectioned and stained immunohistochemically for ASS. Specific staining was observed in the arteries of control mice (Figure 1A), but was absent in arteries from Ass-KO^Tie2^ mice (Figure 1C). The presence of intact endothelium was confirmed by H&E staining (Figures 1B, D).

### Plasma arginine concentrations in male control and Ass-KO^Tie2^ mice

ASS catalyzes the conversion of citrulline to argininosuccinate, the rate-determining step in arginine resynthesis from citrulline. To evaluate the effect of endothelial *Ass* gene deletion on plasma concentrations of arginine and citrulline, plasma amino-acid profiles were determined in healthy and diabetic control and Ass-KO^Tie2^ mice. Arginine and citrulline concentrations were similar in healthy control (117±10 and 56±4 µmol/L, resp.) and Ass-KO^Tie2^ (127±6 and 62±3 µmol/L, resp.). Similar results were also observed in diabetic control (128±12 and 68±5 µmol/L, resp.) and Ass-KO^Tie2^ (129±21 and 73±11 µmol/L, resp.) (Table S2). This suggests that the gene ablation did not result in a major disturbance of circulating arginine and citrulline concentrations.

### Hemodynamics in control and Ass-KO^Tie2^ mice

**Figure 2:**
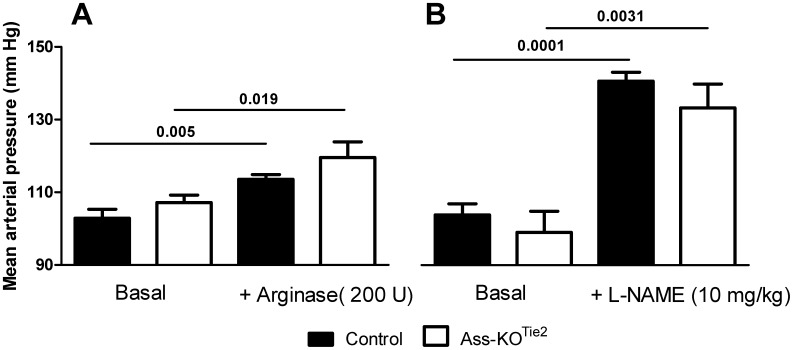
The effect of endothelium-specific *Ass* deletion on hemodynamics of 34-week-old conscious male mice. Black bar: control mice; white bar: Ass-KO^Tie2^ mice. Blood pressure was measured in the same mice 2 (panel A) and 3 days (panel B) after catheterization via a femoral artery catheter connected to a pressure transducer. Panel A: mean arterial pressure (MAP) in the basal condition (left) and after a bolus infusion of 200 U bovine arginase 1 via a jugular vein catheter (right). Panel B: mean arterial pressure in the basal condition (left) and after intravenous L-NAME (10 mg/kg) infusion (right). Values are means ± SEM (control animals: arginase 1: n = 7, L-NAME: n = 5; Ass-KO^Tie2^ mice: arginase 1: n = 5, L-NAME: n = 4; due to loss of catheter patency, numbers were lower on the 3^rd^ day). Note that the Y-axis starts at 90 mm Hg.

To evaluate the effect of endothelial *Ass* deletion on hemodynamics, mean arterial pressure was recorded in conscious male mice as described in Materials and Methods. MAP did not differ between male control (102±2 mmHg) and Ass-KO^Tie2^ (107±3 mmHg) mice (Figure 2A). To assess the role of circulating arginine in blood pressure maintenance, 34-week-old control mice received an intravenous bolus of 200 U arginase 1, which resulted in a rapid decrease of the circulating arginine concentration to ∼13% of the original plasma concentration (84 µmol/L). The lowest arginine concentration was achieved within 10 minutes and the concentration remained at this low level for at least 20 minutes (as determined beforehand in 12-week-old male control mice; Figure S1). This led to a similar (P = 0.66), significant increase of MAP in control (+10±3 mmHg) and Ass-KO^Tie2^ (+12±3 mmHg) mice (Figure 2A). A comparable increase was found in a single female Ass-KO^Tie2^ mouse (MAP 98 and 116 mm Hg under basal conditions and after arginase 1 treatment, respectively). In comparison, a bolus injection of the NOS inhibitor L-NAME (10 mg/kg) resulted in a threefold larger increase of MAP in both control (+37±3 mmHg) and Ass-KO^Tie2^ (+34±1 mmHg) male mice (difference between genotypes: P = 0.42; Figure 2B). These data show that in healthy mice, circulating arginine is essential for blood pressure maintenance.

### Contractile reactivity of control and Ass-KO^Tie2^ arteries *in vitro*

To assess the effects of *Ass* gene ablation on vasomotor responses *in vitro*, we characterized the contractile responses of muscular resistance arteries. Saphenous arteries of male control and Ass-KO^Tie2^ mice at 12 and 34 weeks of age were isolated and subjected to wire myography. The maximal contractile response to 10 µM NA was comparable in control and Ass-KO^Tie2^ mice (Table S2) in both age groups. Furthermore, the sensitivity (pEC_50_ (−log M), Table S2) and maximal contraction (E_max_) to PHE (0.01–10 µM) or K^+^ (40 mM) in the absence or presence of NOS- and cyclooxygenase inhibitors were similar in all groups (Table S2). The lack of arginine resynthesis did not affect contractile responses. Another group of mice was then rendered diabetic by streptozotocin injections to assess the role of arginine resynthesis under pathological conditions. Contractile responses again did not differ between male diabetic control and diabetic Ass-KO^Tie2^ mice (Table S2).

### Relaxing responses to ACh during PHE-induced contraction

**Figure 3:**
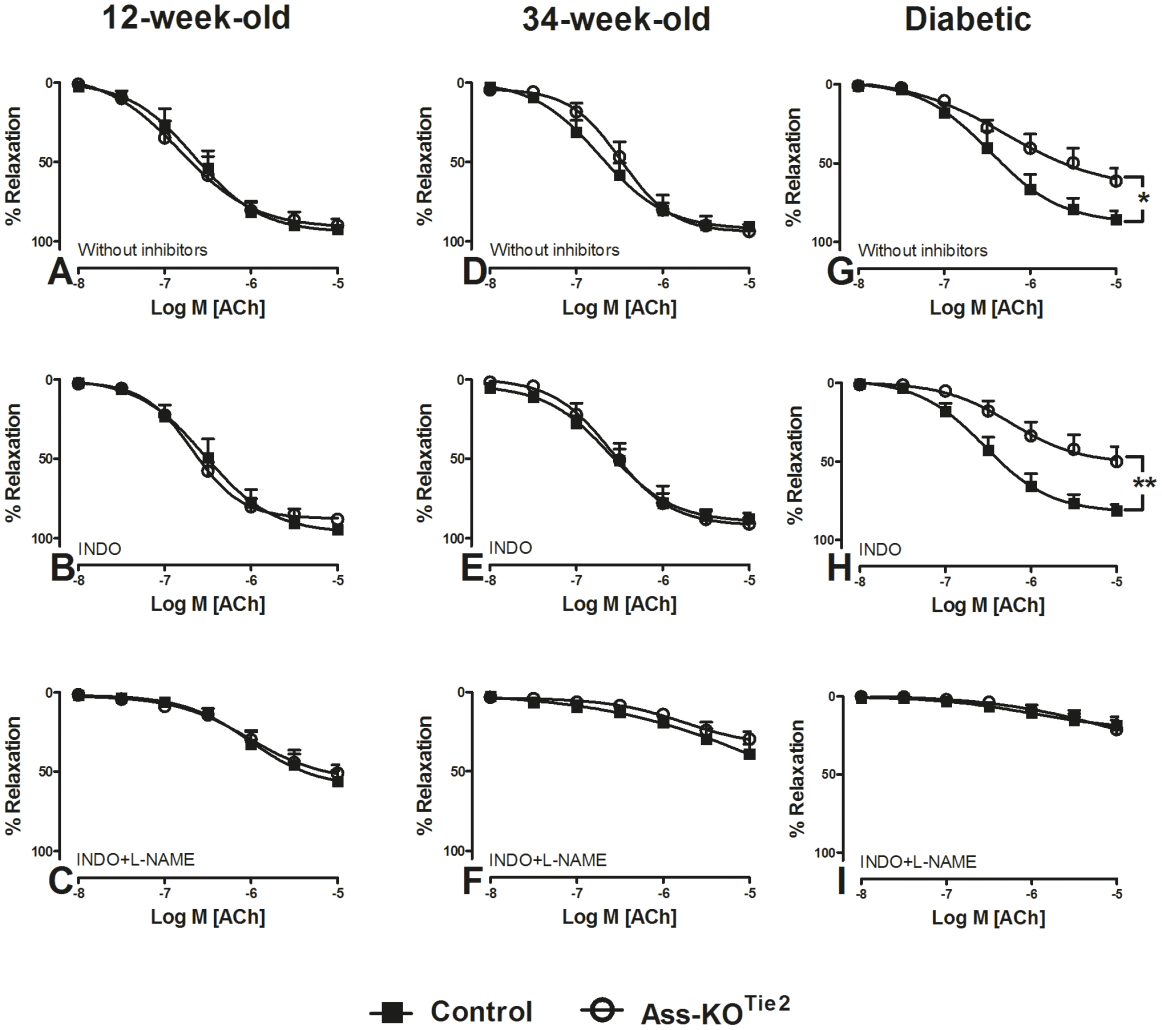
The effect of endothelium-specific *Ass* deletion on relaxation responses of saphenous arteries of healthy and diabetic male mice. Relaxation of PHE (10 µM)-pre-contracted saphenous arteries of 12- (panels A–C) and 34-week-old (panels D–F) healthy and 22-week-old diabetic (panels G–I) male mice to ACh (0.01–10 µM) was determined by wire myography. Black squares: control mice; white circles: Ass-KO^Tie2^ mice. Panels (A, D, G): relaxation responses in the absence of pharmacological inhibitors. Panels (B, E, H): relaxation responses in the presence of INDO (10 µM). Panels (C, F, I): relaxation responses in the presence of both INDO (10 µM) and L-NAME (100 µM). Values are shown as means ± SEM (n = 5–7; for the number of animals per individual experiment see Table 1). *P<0.05 vs. the control, **P<0.01 vs. the control (unpaired t-test).

We then characterized relaxation responses to ACh in healthy and diabetic Ass-KO^Tie2^ and age-matched control mice to assess whether resynthesis of arginine from citrulline may become more important in compromised vessels. E_max_ and pEC_50_ to ACh were similar in healthy control and Ass-KO^Tie2^ (92±2% and 6.6±0.1 versus 90±4% and 6.6±0.1) male mice at 12- weeks of age in the absence (Figure 3A; Table 1) and presence of INDO (Figure 3B, Table 1). Similar results were also observed in 34-week–old male and female mice of both age groups (Figures 3D-E and Figure S2; Table 1; Table S3). In male diabetic Ass-KO^Tie2^ mice, however, the E_max_ to ACh was significantly lower (61±8%) than in diabetic control mice (86±6%) in the absence (Figure 3G; Table 1) and presence of INDO (51±9 and 81±4, resp.; Figure 3H; Table 1). In the presence of both INDO and L-NAME, however, relaxation was suppressed to a similar degree in both healthy and diabetic control and Ass-KO^Tie2^ mice (Figures 3C, F and I; Table 1). Female mice subjected to the same STZ protocol as male mice only temporarily developed elevated blood glucose concentrations, but by 10 weeks after the last STZ treatment, blood glucose was back to normal concentrations (see Table S2). We, nevertheless, measured vascular relaxation in 3 control and 3 Ass-KO^Tie2^ female mice (Figure S2, G–I) and observed no difference between control and STZ-treated mice (P = 0.294 for diabetic control versus diabetic Ass-KO^Tie2^ mice without inhibitors and P = 0.233 in the presence of INDO). We conclude from these data that impaired endothelial arginine resynthesis is responsible for the diminished endothelium-dependent relaxation in male diabetic Ass-KO^Tie2^ mice.

**Table 1:** Effect of endothelium-specific *Ass* deletion on relaxation responses in male mice.

		Control			Ass-KO^Tie2^	
	pEC_50_	E_max_%	n	pEC_50_	E_max_%	n
**12-week-old mice**						
Without inhibitors	6.6±0.1	92±2	6	6.6. ±0.1	90±4	5
INDO	6.5±0.1	94±1	6	6.7±0.1	88±3	7
INDO+L-NAME	6.0±0.1	50±5	7	6.0±0.1	56±7	7
Relaxation to SNP	7.2±0.1	97±1	5	7.1±0.1	96±6	6
Relaxation to EDNO	6.1±0.1	60±4	6	6.3±0.1	60±4	6
**34-week-old mice**						
Without inhibitors	6.7±0.1	90±3	6	6.5±0.1	94±4	5
INDO	6.6±0.1	87±3	6	6.5±0.1	91±4	6
INDO+L-NAME	n.d.	38±6	5	n.d.	30±4	5
Relaxation to SNP	7.2±0.1	97±1	4	7.0±0.2	98±1	5
Relaxation to EDNO	6.1±0.2	56±6	5	5.9±0.1	55±3	7
**22-week-old diabetic mice**						
Without inhibitors	6.5±0.1	86±6	7	6.2±0.2	61±8*	5
INDO	6.5±0.1	81±4	8	6.2±0.2	51±9**	5
INDO+L-NAME	n.d.	18±5	7	n.d.	21±6	5
Relaxation to SNP	6.9±0.1	98±1	5	6.7±0.1	96±1	6
Relaxation to EDNO	6.2±0.1	49±2	6	6.0±0.2	35±4**	4

E_max_ is expressed as % reduction of the maximal contractile response to 10 µM PHE except for EDNO responses (% reduction of maximal contractile response to 40 mM K^+^). All values are shown as mean ± SEM.

**P<0.01 compared to arteries of control mice under the same condition.

*P<0.05 compared to arteries of control mice under the same condition (unpaired t-test). n.d.: not determined.

### Endothelium-derived NO

**Figure 4:**
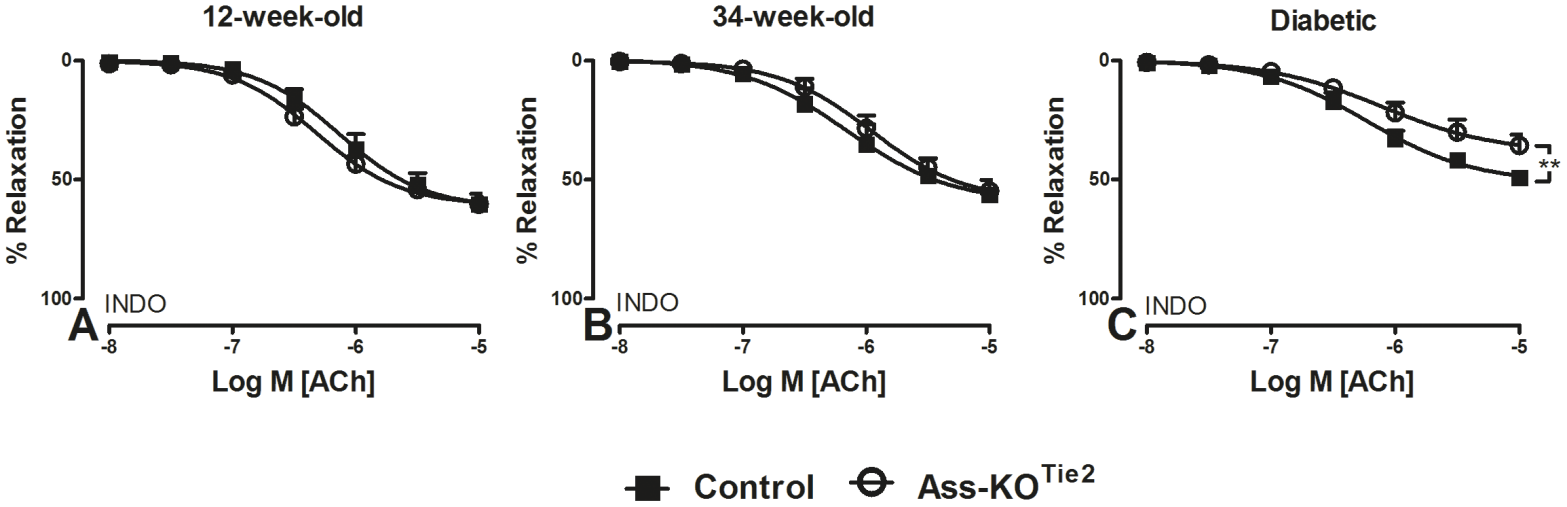
The effect of endothelium-specific *Ass* deletion on relaxation responses of saphenous arteries of healthy and diabetic male mice. Relaxation of K^+^ (40 mM)-pre-contracted saphenous arteries of 12- (panel A) and 34-week-old (panel B) healthy and 22-week-old diabetic (panel C) male mice to ACh (0.01–10 µM) was determined by wire myography. Black squares: control mice; white circles: Ass-KO^Tie2^. All arteries were treated with INDO (10 µM). Values are shown as means ± SEM (n = 4–8; for the number of animals per individual experiment, see Table 1). **P<0.01 vs. control (unpaired t-test).

To evaluate the contribution of endothelium-derived NO in vascular relaxation, we inhibited EDH-mediated relaxations by depolarizing the vessels with high potassium buffer ([K^+^] = 40 mM) and inhibited cyclooxygenases with INDO [22]. Maximal relaxations to ACh were comparable in healthy control and Ass-KO^Tie2^ mice of both age groups (Figures 4A, B; Table 1). In diabetic mice, however, E_max_ to ACh was significantly lower in Ass-KO^Tie2^ (35±4%) than in control mice (49±2%) (P = 0.008; Figure 4C; Table 1). This shows that EDNO-dependent relaxation does not require arginine resynthesis in vessels of healthy mice, whereas NO production relies at least partially on arginine resynthesis in vessels of diabetic mice.

### Relaxing responses to SNP

**Figure 5:**
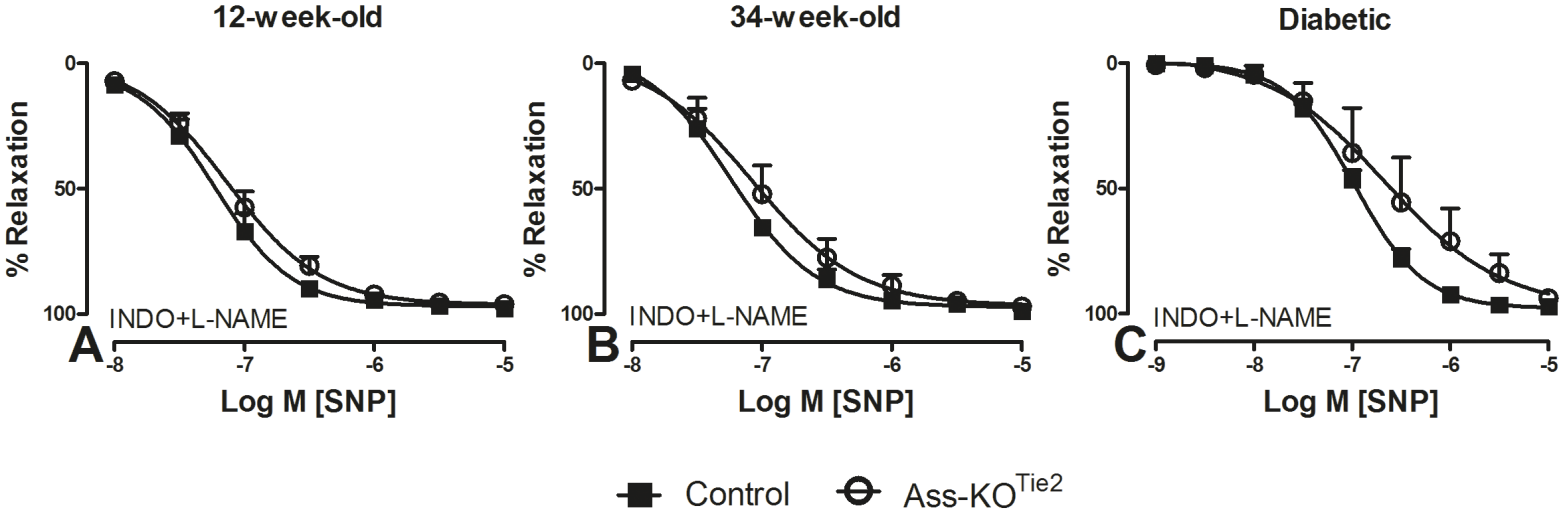
The effect of endothelium-specific *Ass* deletion on relaxation responses of saphenous arteries to sodium nitroprusside. Relaxation of PHE pre-contracted (10 µM) saphenous arteries of 12- (panel A) and 34-week-old healthy (panel B) and 22-week-old diabetic (C) male mice to SNP (0.01–10 µM) was determined by wire myography. Black squares: control mice; white circles: Ass-KO^Tie2^. All experiments were performed in the presence of L-NAME (100 µM) and INDO (10 µM). Values are means ± SEM (n = 5–7; for the number of animals per individual experiment, see Table 1).

To confirm that the responses of the vascular smooth muscle cells were not affected by the genetic manipulation, we blocked endothelial NO production and measured endothelium-independent relaxation in response to an NO donor. PHE-contracted arteries were treated with L-NAME (100 µM) and INDO to block the production of NO and prostaglandins, respectively. Subsequently, the relaxing response to the NO-donor SNP (0.01–10 µM) was measured. pEC_50_ and E_max_ to SNP were comparable in vessels of healthy (Figure 5A, B and Figure S3; Table 1) and diabetic (Figures 5C; Table 1) control and Ass-KO^Tie2^ mice. Relaxing responses to the endothelium-independent NO donor SNP were not affected by genotype, age, or diabetes, indicating that the sensitivity of the vascular smooth muscle cells to NO was unchanged.

## Discussion

In the present study, we evaluated whether deficient arginine resynthesis via endothelial ASS predisposes to endothelial dysfunction. In addition, we addressed the question whether deficient arginine resynthesis aggravates endothelial dysfunction in diabetes. The major finding of this study is that endothelium-dependent relaxations were clearly diminished by endothelial ASS deficiency in diabetic mice, indicating that arginine resynthesis is required to maintain NO production in such compromised vessels. In healthy mice, however, elimination of the *Ass* gene did not influence vasomotor responses or hemodynamic parameters. Apparently, arginine resynthesis is not rate-limiting for NO production in the endothelium of healthy arteries.

We used Tie2 as promoter for the *Cre* gene to delete the floxed *Ass* allele in endothelial cells. It is well established that the Tie2 promoter-enhancer is active in endothelial cells and early hematopoietic precursors [28], resulting in the ablation of the floxed allele in erythrocytes, macrophages, B-cells and T-cells. We, however, never observed ASS protein expression in erythrocytes or lymphocytes of control mice, which makes an effect of deletion of the *Ass* gene in these cells in our experiments unlikely. Expression of *Ass* in macrophages has been reported [29], but saphenous arteries of diabetic mice did not show inflammatory changes or ASS-positive cells in their vascular walls (Figure S4 G, H). Based on these findings, it is unlikely that the presence or absence of ASS protein in macrophages or other hematopoietic cells affected our data.

Blood pressure was recorded in unrestrained mice to assess the effect of ASS deficiency on hemodynamics. Baseline blood pressure values did not differ between control and knockout mice. In addition, L-NAME-induced blood-pressure increases were similar in both groups, suggesting that the contribution of NO to hemodynamics was not affected by ASS deficiency. We used intra-arterial arginase 1 infusion to address the question to what extent plasma arginine contributes to blood pressure regulation. As expected, arginase 1 infusion drastically reduced the plasma arginine concentration and led to a small, but significant increase of MAP. This finding, which appears to reflect the essence of the “arginine paradox” [13], implies that endothelial NO production declines under this condition, because endothelial arginine consumption exceeds its supply or because NOS3 activity is rapidly inactivated in an [arginine]-dependent way. However, the observed increase in MAP after arginine depletion was much smaller than that induced by inhibition of NOS by L-NAME infusion. These findings show that plasma arginine concentration is a determinant of blood pressure, but also that endothelial cells have alternative arginine sources for NO generation.

We used wire myography to study the role of endothelial arginine resynthesis in NO-mediated endothelium-dependent vasodilatation in saphenous arteries. In our previous work, we showed that the relaxation responses in these arteries depend on NO and EDH [22]. In addition, we showed that the contribution of these relaxing factors changed with age. In the present study, we compared the contribution of relaxing factors in 12- and 34-week-old Ass-KO^Tie2^ and control mice and did not find differences in the relaxation responses of healthy mice of both genotypes. Interestingly and consistent with other studies [30], the relaxation responses mediated by EDH were reduced in diabetic mice compared to healthy mice. We used the classical KRB buffer that does not contain arginine to focus on the contribution of resynthesized arginine to NO production. NO-mediated relaxations were significantly reduced in diabetic Ass-KO^Tie2^ mice when compared to diabetic control mice. Since all relaxation differences between control and Ass-KO^Tie2^ mice were abolished by the presence of L-NAME, they were not due to the effects of ASS deficiency on EDH-mediated relaxations. In addition, SNP-induced relaxations displayed similar pEC_50_ and E_max_ in both genotypes. We also did not find quantitative differences in the response to SNP between diabetic control and diabetic Ass-KO^Tie2^ mice. The difference between control and Ass-KO^Tie2^ mice was, therefore, not due to an altered sensitivity of smooth muscle cells to NO. We have considered carrying out experiments on diabetic mice supplemented with arginine and myograph experiments with isolated arteries from Ass-KO^Tie2^ mice in the presence of arginine. In principle, both interventions should reverse the effect of deficient arginine recycling. However, because our recent studies showed that intravascular arginine supplementation does not increase intracellular arginine availability and that, instead, intravascular citrulline is the supplementation of choice [31], we did not carry out such experiments. Further support comes from a recent publication in Hypertension that demonstrated that supplementation with L-citrulline was more effective in preventing glomerular hyperfiltration and proteinuria in diabetic rats than L-arginine supplementation, even though both increased plasma L-arginine concentrations [32].

The difference in the magnitude of the effect of intravenous arginase 1 infusion and L-NAME administration shows that arterial endothelium can use arginine from several different arginine pools. These pools, which include intracellular arginine that is resynthesized from citrulline or released from protein breakdown, and extracellular arginine imported via specific transporters, may be available to NOS3 under different circumstances [33], [34]. In healthy arteries, ASS deficiency apparently does not lead to profound endothelial dysfunction because of extensive functional redundancy of the arginine pools. In arteries of diabetic mice, however, we observed that ASS deficiency reduced NO-mediated endothelium-dependent relaxations. It was reported earlier that diabetes attenuates the endothelium-dependent relaxation responses and increases expression and activity of arginases in the aortic wall [33]. However, we did not observe arginase 1 or arginase 2 expression in diabetic saphenous arteries (Figure S4 A–F). One should keep in mind that since arginases have a very high catalytic activity, amounts that are not detectable by immunohistochemistry could still represent physiologically relevant activities. STZ-induced diabetes was shown to decrease expression of the arginine transporter CAT1 in the kidney [35]. Although a similar effect of diabetes on CAT1 in saphenous artery endothelium has not been reported thus far, downregulation of arginine transporter(s) may contribute to the observed dependence on arginine resynthesis in diabetes to maintain adequate intracellular arginine availability for NOS3. Whether or not endothelial protein degradation is enhanced in diabetic mice remains to be sorted out [36]–[38], but even if it is increased, it can probably not affect arginine availability under the long-term steady state circumstances that we used in the current experiments.

An aspect that requires attention in future studies is that endothelial cells in intact resistance arteries are coupled to smooth muscle cells via gap junctions [39]. These proteins allow for diffusion of small molecules (<1000 Da), including free amino acids, from one cell to another [40]. It is therefore conceivable that the smooth muscle cells in arteries from healthy mice represent an arginine reservoir for endothelial cells. In endothelial cells, gap junctions are primarily formed of connexins proteins CX37, CX40 and CX43. Interestingly, their expression is reduced in vascular walls of diabetic mice [41], [42]. Unfortunately, it is technically challenging to establish whether a gap junction-dependent arginine flux contributes to the maintenance of intra-endothelial arginine concentration. Firstly, Cx43 deficiency is neonatally lethal [43] and secondly, both Cx40 [24] and Cx37 [44] have a direct interaction with NOS3, with Cx37 deficiency even increasing NO production in vitro [44]. Pharmacological tools, such as carbenoxolone and heptanol, are notoriously non-selective [45], while the applicability of the “GAP” peptides cocktail in vivo and their specificity with respect to the homo- and hetero-cellular communication still need to be explored [46]. Although the aforementioned issues complicate the firm establishment of a role for gap junctions in arginine bioavailability in the endothelium, we speculate that diabetic Ass-KO^Tie2^ mice display endothelial dysfunction due to a decreased gap junction-dependent arginine flux.

The concentration of intra-endothelial arginine may also indirectly affect the production of NO. Previous studies showed that arginine supplementation increases the transcription of GTP cyclohydrolase 1 in diabetic rats [47]. GTP cyclohydrolase 1, the first enzyme in the *de novo* synthesis of BH4, elevates the intracellular concentration of BH4 which is a necessary cofactor for NOS3 activity [47]. In our diabetic Ass-KO^Tie2^ mice, impaired resynthesis of arginine could be responsible for the uncoupling of NOS3 due to reduced BH4 production, but this notion needs to be investigated further.

In summary, the present study shows that deletion of the floxed *Ass* gene with Cre recombinase under the control of Tie2-cre promoter does not affect MAP or heart rate in healthy mice. In addition, *in vitro* studies of isolated saphenous arteries showed that, in healthy mice, relaxation responses were unaffected by the ablation of the *Ass* gene. In diabetic mice, however, ablation of *Ass* resulted in diminished endothelium-derived NO-mediated vascular relaxation responses. These results are exciting, since they suggest that diabetic patients suffering from endothelial dysfunction may benefit from therapies focusing on either increasing ASS activity or boosting intracellular arginine levels. In this respect it is interesting to note that *Ass* gene expression is diminished in STZ-treated rats and that insulin treatment upregulates ASS transcription in these animals [48].

## Supporting Information

Figure S1 **Change in plasma arginine concentrations after intravenous arginase 1 infusion (200 U) in 12-week-old control (** ***Ass^fl/fl^)*** **mice.**
(PPTX)

Figure S2 **The effect of endothelium-specific** ***Ass*** **deletion on relaxation responses in healthy and diabetic female mice.** Saphenous arteries of 12- (A–C) and 34-week-old (D–F) healthy and 22-week-old diabetic (panels G–I) female mice were pre-contracted with PHE (10 µM) and relaxation responses to ACh (0.01–10 µM) were determined by wire myography. Black squares: control mice; white circles: Ass-KO^Tie2^ mice. Panels (A, D, G): in the absence of pharmacological inhibitors. Panels (B, E, H): in the presence of INDO (10 µM). Panels (C, F, I): in the presence of both INDO (10 µM) and L-NAME (100 µM). Values are shown as means ± SEM (n = 5 for healthy mice; n = 3 for diabetic mice).
(PPTX)

Figure S3 **The effect of endothelium-specific** ***Ass*** **deletion on relaxation responses to sodium nitroprusside in female mice.** Saphenous arteries of 12- (A) and 34-week-old (B) female mice were pre-contracted with PHE (10 µM) and relaxation responses to SNP (0.01–10 µM) were determined by wire myography. Black squares: control mice; white circles: Ass-KO^Tie2^. All experiments were performed in the presence of L-NAME (100 µM) and INDO (10 µM). Values are means ± SEM (n = 5).
(PPTX)

Figure S4 **Immunohistochemical staining for the presence of arginase 1, -2 and ASS in the walls of saphenous arteries of diabetic mice.** Panels A and D represent staining for arginase 1 and 2, respectively. Note the absence of arginase 1 and -2 positive cells both in the endothelium and the media/adventitia. Panels B and E represent the negative controls for arginase 1 and -2, respectively. Panels C and F show positive controls for arginase 1 (liver) and arginase 2 (kidney cortex). Note that plasma proteins do cause background staining for arginase 1. Panel G shows ASS staining of the endothelium, but no ASS-positive cells in the tunica media. Panel H shows an H&E staining of the vessel shown in panel G to demonstrate absence of inflammatory changes. Bar = 10 µm for all panels.
(PPTX)

Table S1 **Fasting plasma blood glucose concentrations in male and female control and Ass-KO^Tie2^ mice before and after streptozotocin treatment.** Mice were fasted for 4 hours before blood glucose was measured before or 1, 4, or 10 weeks after the last STZ injection. Data are shown as mean ± SEM (n = 5 for STZ-treated mice). Note that basal blood glucose values for male and female control mice were taken from 12- to 15-week-old C57BL/6J wild type mice in another experiment. Basal values for Ass-KO^Tie2^ mice (12-week–old) are from this series of experiments.
(DOCX)

Table S2 **Effect of** ***Ass*** **gene deletion on plasma amino acid concentrations, saphenous artery diameter and contractile responses in male mice.** E_max_ values are expressed as % of the maximal response to noradrenaline (NA; 10 µM). All values are shown as mean ± SEM. n.d. = not determined.
(DOC)

Table S3 **Effect of endothelium-specific deletion of ASS on relaxation responses in female mice.** E_max_ expressed as % reduction of the maximal contractile response to 10 µM PHE. All values are shown as mean ± SEM. n.d: not determined.
(DOCX)

## References

[1] Tsihlis ND, Oustwani CS, Vavra AK, Jiang Q, Keefer LK, et al (2011) Nitric oxide inhibits vascular smooth muscle cell proliferation and neointimal hyperplasia by increasing the ubiquitination and degradation of UbcH10. Cell Biochem Biophys 60: 89–97. 21448667 10.1007/s12013-011-9179-3 PMC6959532

[2] Radomski MW, Palmer RM, Moncada S (1987) Endogenous nitric oxide inhibits human platelet adhesion to vascular endothelium. Lancet 2: 1057–1058. 2889967 10.1016/s0140-6736(87)91481-4

[3] Wang GR, Zhu Y, Halushka PV, Lincoln TM, Mendelsohn ME (1998) Mechanism of platelet inhibition by nitric oxide: in vivo phosphorylation of thromboxane receptor by cyclic GMP-dependent protein kinase. Proc Natl Acad Sci U S A 95: 4888–4893. 9560198 10.1073/pnas.95.9.4888 PMC20183

[4] Hogg N, Kalyanaraman B, Joseph J, Struck A, Parthasarathy S (1993) Inhibition of low-density lipoprotein oxidation by nitric oxide. Potential role in atherogenesis. FEBS Lett 334: 170–174. 8224243 10.1016/0014-5793(93)81706-6

[5] Taddei S, Virdis A, Mattei P, Ghiadoni L, Sudano I, et al (1996) Defective L-arginine-nitric oxide pathway in offspring of essential hypertensive patients. Circulation 94: 1298–1303. 8822983 10.1161/01.cir.94.6.1298

[6] Elkayam U, Khan S, Mehboob A, Ahsan N (2002) Impaired endothelium-mediated vasodilation in heart failure: clinical evidence and the potential for therapy. J Card Fail 8: 15–20. 11862578 10.1054/jcaf.2002.31910

[7] Feron O, Dessy C, Moniotte S, Desager JP, Balligand JL (1999) Hypercholesterolemia decreases nitric oxide production by promoting the interaction of caveolin and endothelial nitric oxide synthase. J Clin Invest 103: 897–905. 10079111 10.1172/JCI4829 PMC408139

[8] Li H, Forstermann U (2009) Prevention of atherosclerosis by interference with the vascular nitric oxide system. Curr Pharm Des 15: 3133–3145. 19754387 10.2174/138161209789058002

[9] Fukao M, Hattori Y, Kanno M, Sakuma I, Kitabatake A (1997) Alterations in endothelium-dependent hyperpolarization and relaxation in mesenteric arteries from streptozotocin-induced diabetic rats. Br J Pharmacol 121: 1383–1391. 9257918 10.1038/sj.bjp.0701258 PMC1564820

[10] Gold ME, Bush PA, Ignarro LJ (1989) Depletion of arterial L-arginine causes reversible tolerance to endothelium-dependent relaxation. Biochem Biophys Res Commun 164: 714–721. 2510722 10.1016/0006-291x(89)91518-0

[11] Baydoun AR, Emery PW, Pearson JD, Mann GE (1990) Substrate-dependent regulation of intracellular amino acid concentrations in cultured bovine aortic endothelial cells. Biochem Biophys Res Commun 173: 940–948. 2268354 10.1016/s0006-291x(05)80876-9

[12] Lerman A, Burnett JC Jr, Higano ST, McKinley LJ, Holmes DR Jr (1998) Long-term L-arginine supplementation improves small-vessel coronary endothelial function in humans. Circulation 97: 2123–2128. 9626172 10.1161/01.cir.97.21.2123

[13] Kurz S, Harrison DG (1997) Insulin and the arginine paradox. J Clin Invest 99: 369–370. 9022065 10.1172/JCI119166 PMC507805

[14] Vukosavljevic N, Jaron D, Barbee KA, Buerk DG (2006) Quantifying the L-arginine paradox in vivo. Microvasc Res 71: 48–54. 16316668 10.1016/j.mvr.2005.10.006

[15] Takabe W, Kanai Y, Chairoungdua A, Shibata N, Toi S, et al (2004) Lysophosphatidylcholine enhances cytokine production of endothelial cells via induction of L-type amino acid transporter 1 and cell surface antigen 4F2. Arterioscler Thromb Vasc Biol 24: 1640–1645. 15178563 10.1161/01.ATV.0000134377.17680.26

[16] Wu G, Morris SM Jr (1998) Arginine metabolism: nitric oxide and beyond. Biochem J 336 (Pt 1): 1–17. 9806879 10.1042/bj3360001 PMC1219836

[17] Chicoine LG, Paffett ML, Young TL, Nelin LD (2004) Arginase inhibition increases nitric oxide production in bovine pulmonary arterial endothelial cells. Am J Physiol Lung Cell Mol Physiol 287: L60–68. 14977627 10.1152/ajplung.00194.2003

[18] Zhang C, Hein TW, Wang W, Chang CI, Kuo L (2001) Constitutive expression of arginase in microvascular endothelial cells counteracts nitric oxide-mediated vasodilatory function. FASEB J 15: 1264–1266. 11344108 10.1096/fj.00-0681fje

[19] Romero MJ, Platt DH, Tawfik HE, Labazi M, El-Remessy AB, et al (2008) Diabetes-induced coronary vascular dysfunction involves increased arginase activity. Circ Res 102: 95–102. 17967788 10.1161/CIRCRESAHA.107.155028 PMC2822539

[20] Shin WS, Berkowitz DE, Ryoo SW (2012) Increased arginase II activity contributes to endothelial dysfunction through endothelial nitric oxide synthase uncoupling in aged mice. Exp Mol Med 44: 594–602. 22854495 10.3858/emm.2012.44.10.068 PMC3490081

[21] Goodwin BL, Solomonson LP, Eichler DC (2004) Argininosuccinate synthase expression is required to maintain nitric oxide production and cell viability in aortic endothelial cells. J Biol Chem 279: 18353–18360. 14970240 10.1074/jbc.M308160200

[22] Chennupati R, Lamers WH, Koehler SE, De Mey JG (2013) Endothelium-dependent hyperpolarization-related relaxations diminish with age in murine saphenous arteries of both sexes. Br J Pharmacol 169: 1486–1499. 23488619 10.1111/bph.12175 PMC3724106

[23] Marion V, Sankaranarayanan S, de Theije C, van Dijk P, Hakvoort TB, et al (2013) Hepatic adaptation compensates inactivation of intestinal arginine biosynthesis in suckling mice. PLoS One 8: e67021. 23785515 10.1371/journal.pone.0067021 PMC3681768

[24] Alonso F, Boittin FX, Beny JL, Haefliger JA (2010) Loss of connexin40 is associated with decreased endothelium-dependent relaxations and eNOS levels in the mouse aorta. Am J Physiol Heart Circ Physiol 299: H1365–1373. 20802140 10.1152/ajpheart.00029.2010

[25] van Eijk HM, Rooyakkers DR, Deutz NE (1993) Rapid routine determination of amino acids in plasma by high-performance liquid chromatography with a 2–3 microns Spherisorb ODS II column. J Chromatogr 620: 143–148. 8106581 10.1016/0378-4347(93)80062-9

[26] Storkebaum E, Ruiz de Almodovar C, Meens M, Zacchigna S, Mazzone M, et al (2010) Impaired autonomic regulation of resistance arteries in mice with low vascular endothelial growth factor or upon vascular endothelial growth factor trap delivery. Circulation 122: 273–281. 20606119 10.1161/CIRCULATIONAHA.109.929364

[27] Hilgers RH, Janssen GM, Fazzi GE, De Mey JG (2010) Twenty-four-hour exposure to altered blood flow modifies endothelial Ca2+-activated K+ channels in rat mesenteric arteries. J Pharmacol Exp Ther 333: 210–217. 20040579 10.1124/jpet.109.161448

[28] Tang Y, Harrington A, Yang X, Friesel RE, Liaw L (2010) The contribution of the Tie2+ lineage to primitive and definitive hematopoietic cells. Genesis 48: 563–567. 20645309 10.1002/dvg.20654 PMC2944906

[29] Nussler AK, Billiar TR, Liu ZZ, Morris SM Jr (1994) Coinduction of nitric oxide synthase and argininosuccinate synthetase in a murine macrophage cell line. Implications for regulation of nitric oxide production. J Biol Chem 269: 1257–1261. 7507106

[30] Burnham MP, Johnson IT, Weston AH (2006) Impaired small-conductance Ca2+-activated K+ channel-dependent EDHF responses in Type II diabetic ZDF rats. Br J Pharmacol 148: 434–441. 16682967 10.1038/sj.bjp.0706748 PMC1751791

[31] Wijnands KA, Vink H, Briede JJ, van Faassen EE, Lamers WH, et al (2012) Citrulline a more suitable substrate than arginine to restore NO production and the microcirculation during endotoxemia. PLoS One 7: e37439. 22666356 10.1371/journal.pone.0037439 PMC3362574

[32] Persson P, Fasching A, Teerlink T, Hansell P, Palm F (2014) l-Citrulline, But Not l-Arginine, Prevents Diabetes Mellitus-Induced Glomerular Hyperfiltration and Proteinuria in Rat. Hypertension 64: ePub ahead of print; http://hyper.ahajournals.org/content/early/2014/05/27/HYPERTENSIONAHA.114.03519 10.1161/HYPERTENSIONAHA.114.03519 24866144

[33] Toque HA, Nunes KP, Yao L, Xu Z, Kondrikov D, et al (2013) Akita spontaneously type 1 diabetic mice exhibit elevated vascular arginase and impaired vascular endothelial and nitrergic function. PloS one 8: e72277. 23977269 10.1371/journal.pone.0072277 PMC3747112

[34] Karbach S, Simon A, Slenzka A, Jaenecke I, Habermeier A, et al (2011) Relative contribution of different l-arginine sources to the substrate supply of endothelial nitric oxide synthase. J Mol Cell Cardiol 51: 855–861. 21839088 10.1016/j.yjmcc.2011.07.024

[35] Levin-Iaina N, Schwartz I, Chernichovsky T, Davidovitch A, Iaina A, et al (2007) Tubular and glomerular L-arginine transport (uptake and transporters) and the nitric oxide synthases in ischemic acute renal failure (iARF) in streptozotocin-induced diabetic rats (STZ-DM). Renal failure 29: 1031–1038. 18067052 10.1080/08860220701641744

[36] Chen F, Chen B, Xiao FQ, Wu YT, Wang RH, et al (2014) Autophagy Protects Against Senescence and Apoptosis via the RAS-Mitochondria in High-Glucose-Induced Endothelial Cells. Cell Physiol Biochem 33: 1058–1074. 24732710 10.1159/000358676

[37] Queisser MA, Yao D, Geisler S, Hammes HP, Lochnit G, et al (2010) Hyperglycemia impairs proteasome function by methylglyoxal. Diabetes 59: 670–678. 20009088 10.2337/db08-1565 PMC2828656

[38] Xu J, Wang S, Zhang M, Wang Q, Asfa S, et al (2012) Tyrosine nitration of PA700 links proteasome activation to endothelial dysfunction in mouse models with cardiovascular risk factors. PLoS One 7: e29649. 22272240 10.1371/journal.pone.0029649 PMC3260160

[39] Sandow SL, Goto K, Rummery NM, Hill CE (2004) Developmental changes in myoendothelial gap junction mediated vasodilator activity in the rat saphenous artery. J Physiol 556: 875–886. 14766938 10.1113/jphysiol.2003.058669 PMC1665009

[40] Giepmans BN (2004) Gap junctions and connexin-interacting proteins. Cardiovasc Res 62: 233–245. 15094344 10.1016/j.cardiores.2003.12.009

[41] Hou CJ, Tsai CH, Su CH, Wu YJ, Chen SJ, et al (2008) Diabetes reduces aortic endothelial gap junctions in ApoE-deficient mice: simvastatin exacerbates the reduction. J Histochem Cytochem 56: 745–752. 18443364 10.1369/jhc.2008.950816 PMC2443608

[42] Bobbie MW, Roy S, Trudeau K, Munger SJ, Simon AM (2010) Reduced connexin 43 expression and its effect on the development of vascular lesions in retinas of diabetic mice. Invest Ophthalmol Vis Sci 51: 3758–3763. 20130277 10.1167/iovs.09-4489 PMC2904019

[43] Gutstein DE, Morley GE, Tamaddon H, Vaidya D, Schneider MD, et al (2001) Conduction slowing and sudden arrhythmic death in mice with cardiac-restricted inactivation of connexin43. Circ Res 88: 333–339. 11179202 10.1161/01.res.88.3.333 PMC3630465

[44] Pfenniger A, Derouette JP, Verma V, Lin X, Foglia B, et al (2010) Gap junction protein Cx37 interacts with endothelial nitric oxide synthase in endothelial cells. Arterioscler Thromb Vasc Biol 30: 827–834. 20081116 10.1161/ATVBAHA.109.200816 PMC2930827

[45] Matchkov VV, Rahman A, Peng H, Nilsson H, Aalkjaer C (2004) Junctional and nonjunctional effects of heptanol and glycyrrhetinic acid derivates in rat mesenteric small arteries. Br J Pharmacol 142: 961–972. 15210581 10.1038/sj.bjp.0705870 PMC1575116

[46] Dahl G (2007) Gap junction-mimetic peptides do work, but in unexpected ways. Cell Commun Adhes 14: 259–264. 18392993 10.1080/15419060801891018

[47] Wu G, Meininger CJ (2009) Nitric oxide and vascular insulin resistance. Biofactors 35: 21–27. 19319842 10.1002/biof.3

[48] Haines RJ, Corbin KD, Pendleton LC, Meininger CJ, Eichler DC (2012) Insulin transcriptionally regulates argininosuccinate synthase to maintain vascular endothelial function. Biochem Biophys Res Commun 421: 9–14. 22452988 10.1016/j.bbrc.2012.03.074 PMC4164166

