# Supplementary figures and images for: Endothelial Arginine Resynthesis Contributes to the Maintenance of Vasomotor Function in Male Diabetic Mice
Source: PLoS One. 2014 Jul 17;9(7):e102264. doi: 10.1371/journal.pone.0102264 (PMC4102520; doi:10.1371/journal.pone.0102264)

## Slide 1
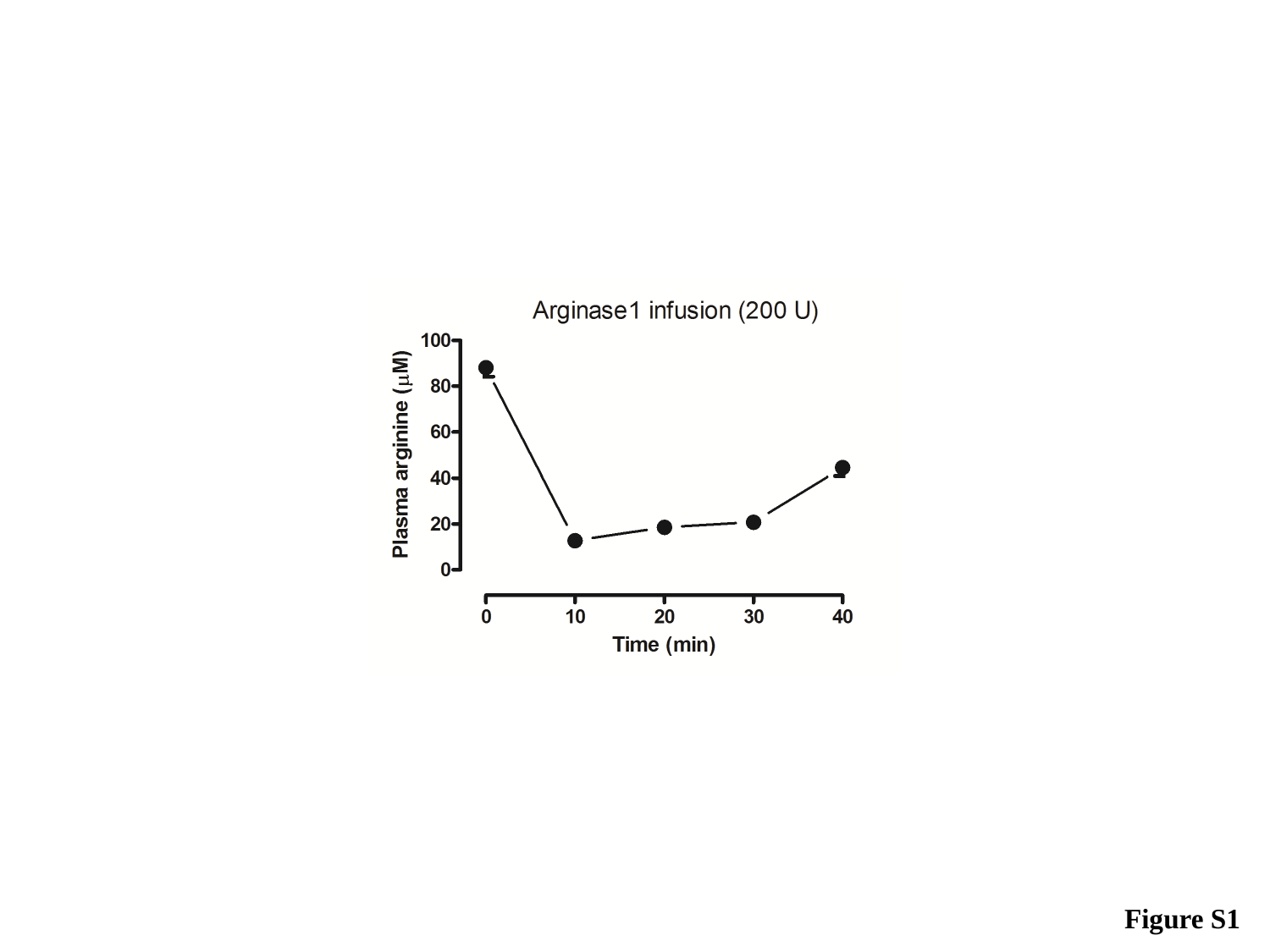

Figure S1

Supplement: Figure S1 — Change in plasma arginine concentrations after intravenous arginase 1 infusion (200 U) in 12-week-old control ( Assfl/fl) mice. (PPTX) [file pone.0102264.s001.pptx]

## Slide 1
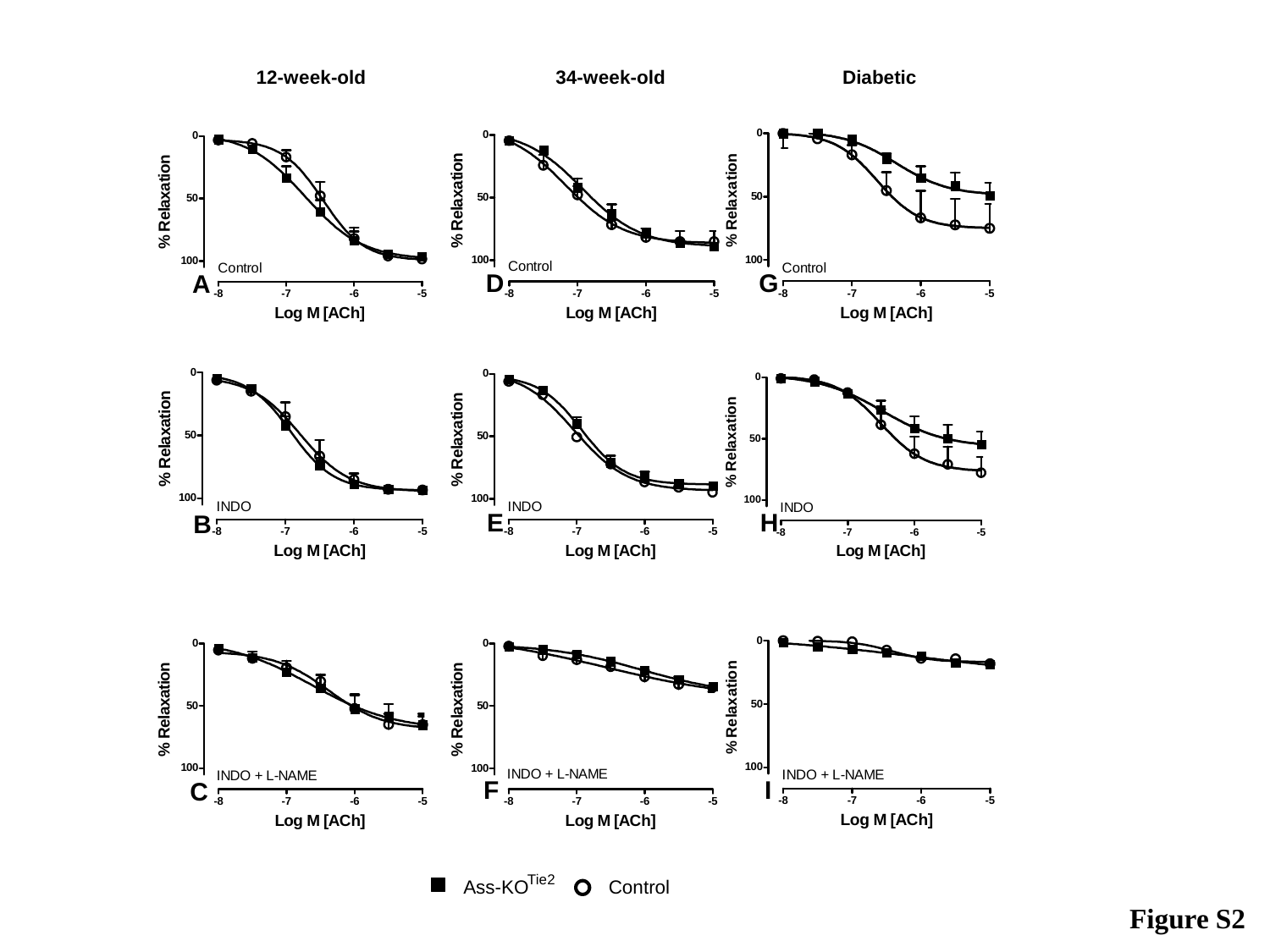

Figure S2

Supplement: Figure S2 — The effect of endothelium-specific Ass deletion on relaxation responses in healthy and diabetic female mice. Saphenous arteries of 12- (A–C) and 34-week-old (D–F) healthy and 22-week-old diabetic (panels G–I) female mice were pre-contracted with PHE (10 µM) and relaxation responses to ACh (0.01–10 µM) were determined by wire myography. Black squares: control mice; white circles: Ass-KOTie2 mice. Panels (A, D, G): in the absence of pharmacological inhibitors. Panels (B, E, H): in the presence of INDO (10 µM). Panels (C, F, I): in the presence of both INDO (10 µM) and L-NAME (100 µM). Values are shown as means ± SEM (n = 5 for healthy mice; n = 3 for diabetic mice). (PPTX) [file pone.0102264.s002.pptx]

## Slide 1
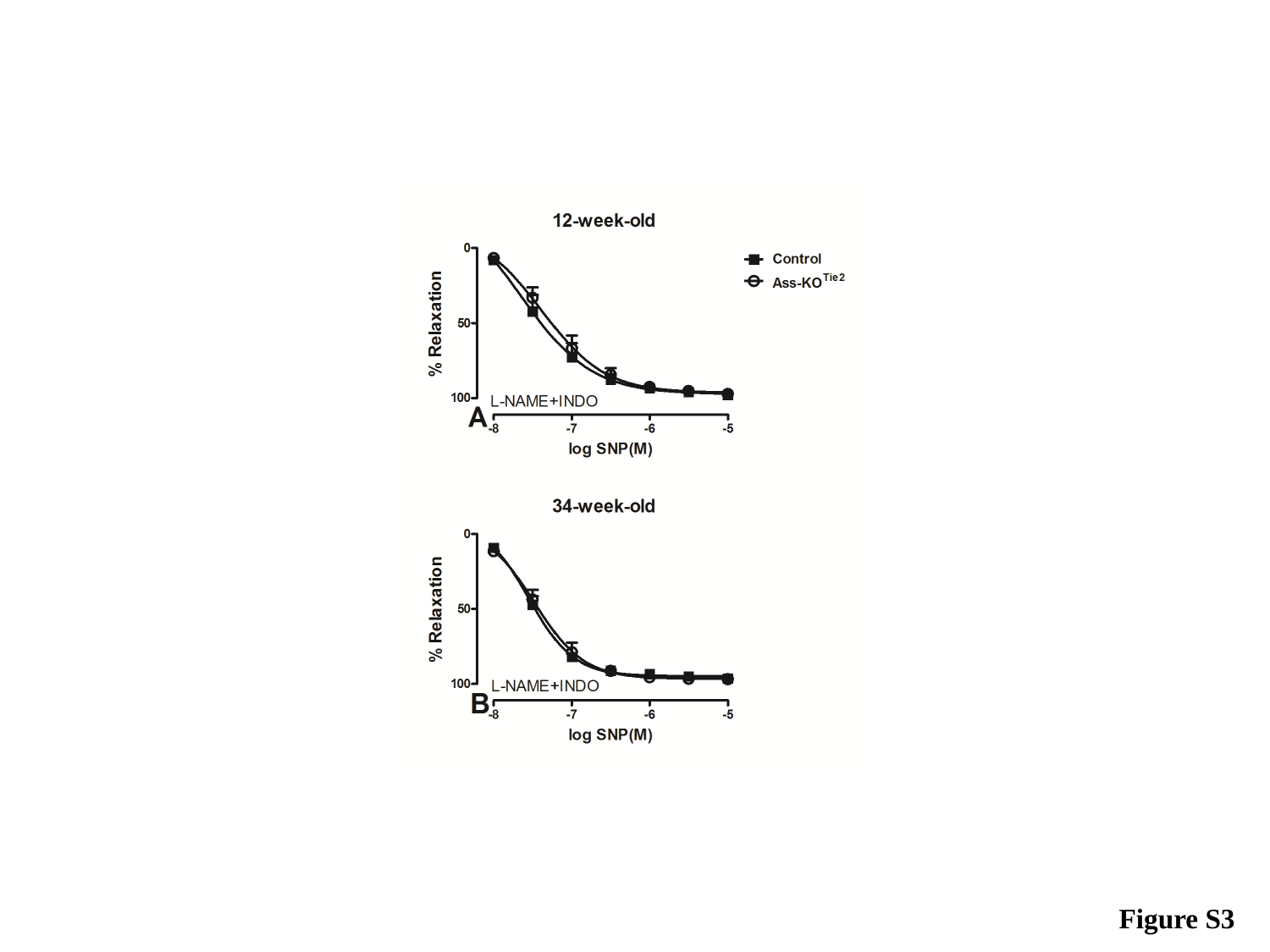

Figure S3

Supplement: Figure S3 — The effect of endothelium-specific Ass deletion on relaxation responses to sodium nitroprusside in female mice. Saphenous arteries of 12- (A) and 34-week-old (B) female mice were pre-contracted with PHE (10 µM) and relaxation responses to SNP (0.01–10 µM) were determined by wire myography. Black squares: control mice; white circles: Ass-KOTie2. All experiments were performed in the presence of L-NAME (100 µM) and INDO (10 µM). Values are means ± SEM (n = 5). (PPTX) [file pone.0102264.s003.pptx]

## Slide 1
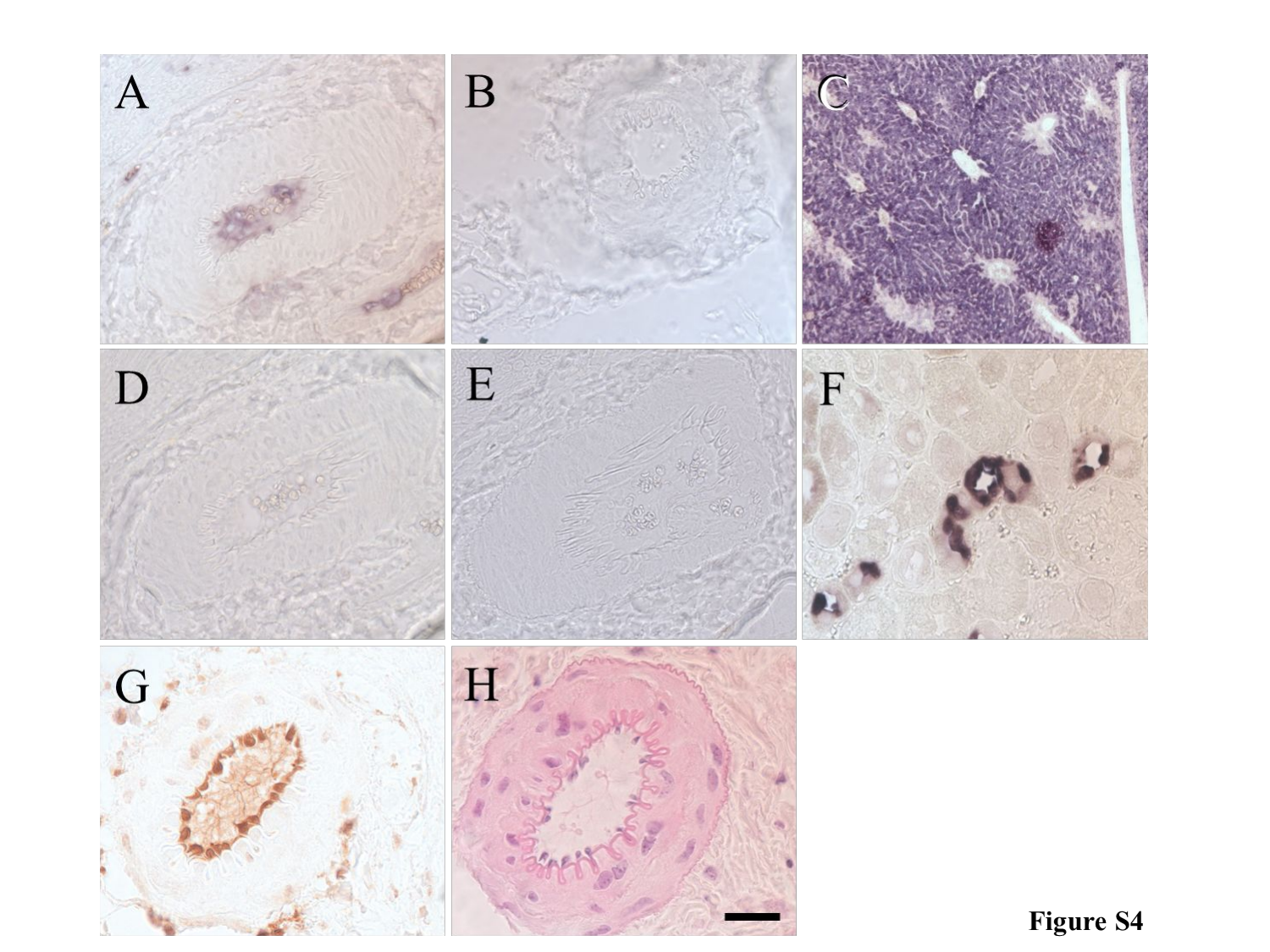

Supplement: Figure S4 — Immunohistochemical staining for the presence of arginase 1, -2 and ASS in the walls of saphenous arteries of diabetic mice. Panels A and D represent staining for arginase 1 and 2, respectively. Note the absence of arginase 1 and -2 positive cells both in the endothelium and the media/adventitia. Panels B and E represent the negative controls for arginase 1 and -2, respectively. Panels C and F show positive controls for arginase 1 (liver) and arginase 2 (kidney cortex). Note that plasma proteins do cause background staining for arginase 1. Panel G shows ASS staining of the endothelium, but no ASS-positive cells in the tunica media. Panel H shows an H&E staining of the vessel shown in panel G to demonstrate absence of inflammatory changes. Bar = 10 µm for all panels. (PPTX) [file pone.0102264.s004.pptx]
