# Supplementary material for: Endothelial Arginine Resynthesis Contributes to the Maintenance of Vasomotor Function in Male Diabetic Mice
Source: PLoS One. 2014 Jul 17;9(7):e102264. doi: 10.1371/journal.pone.0102264 (PMC4102520; doi:10.1371/journal.pone.0102264)
Supplement: Table S1 — Fasting plasma blood glucose concentrations in male and female control and Ass-KOTie2 mice before and after streptozotocin treatment. Mice were fasted for 4 hours before blood glucose was measured before or 1, 4, or 10 weeks after the last STZ injection. Data are shown as mean ± SEM (n = 5 for STZ-treated mice). Note that basal blood glucose values for male and female control mice were taken from 12- to 15-week-old C57BL/6J wild type mice in another experiment. Basal values for Ass-KOTie2 mice (12-week–old) are from this series of experiments. (DOCX) [file pone.0102264.s005.docx]

**Supplemental Table 1: Fasting plasma blood glucose concentrations in male and female control and Ass-KO^Tie2^ mice before and after streptozotocin treatment.**

|  | Fasting blood glucose (mmol/L) | Fasting plasma blood glucose (mmol/L) at the indicated times after the last STZ injection | | |
| --- | --- | --- | --- | --- |
|  | Basal values | 1 week | 4 weeks | 10 weeks |
| **Male mice** |  |  |  |  |
| Control | 8.1 ± 2.9 (n=8) | 20.4 ± 0.9 | 24.2 ± 1.0 | 22.8 ± 0.8 |
| Ass-KO^Tie2^ | 7.1 (n=1) | 19.5 ± 0.8 | 26.1 ± 1.2 | 23.1 ± 2.0 |
| **Female mice** |  |  |  |  |
| Control | 8.9 ± 3.0 (n=9) | 12.1 ± 1.0 | 13.8 ± 1.1 | 7.8 ± 1.0 |
| Ass-KO^Tie2^ | 8.1 ± 0.3 (n=3) | 13.7 ± 0.7 | 13.5 ± 0.9 | 8.4 ± 0.5 |

Mice were fasted for 4 hours before blood glucose was measured before or 1, 4, or 10 weeks after the last STZ injection. Data are shown as mean ± SEM (n= 5 for STZ-treated mice). Note that basal blood glucose values for male and female control mice were taken from 12- to 15-week-old C57BL/6J wild type mice in another experiment. Basal values for Ass-KO^Tie2^ mice (12-week–old) are from this series of experiments.
