# Supplementary material for: Endothelial Arginine Resynthesis Contributes to the Maintenance of Vasomotor Function in Male Diabetic Mice
Source: PLoS One. 2014 Jul 17;9(7):e102264. doi: 10.1371/journal.pone.0102264 (PMC4102520; doi:10.1371/journal.pone.0102264)
Supplement: Table S2 — Effect of Ass gene deletion on plasma amino acid concentrations, saphenous artery diameter and contractile responses in male mice. Emax values are expressed as % of the maximal response to noradrenaline (NA; 10 µM). All values are shown as mean ± SEM. n.d. = not determined. (DOC) [file pone.0102264.s006.doc]

**Table S2:** Effect of *Ass* gene deletion on plasma amino acid concentrations, saphenous artery diameter and contractile responses in male mice.

|  |  | **Control** |  |  | **Ass-KO^Tie2^** |  |
| --- | --- | --- | --- | --- | --- | --- |
| **Plasma amino acids** |  |  |  |  |  |  |
| ***Healthy mice (12-week-old)*** |  |  |  |  |  |  |
| L-arginine |  | 117 ± 10 |  |  | 127 ± 6 |  |
| L-citrulline |  | 56 ± 4 |  |  | 62 ± 3 |  |
|  |  |  |  |  |  |  |
| ***Diabetic mice (22-week-old)*** |  |  |  |  |  |  |
| L-arginine |  | 128 ± 12 |  |  | 129 ± 21 |  |
| L-citrulline |  | 68 ± 5 |  |  | 73 ± 11 |  |
|  |  |  |  |  |  |  |
| **Optimal diameter (µm)** |  |  |  |  |  |  |
| 12-week-old mice |  | 251 ± 3 |  |  | 241 ± 7 |  |
| 34-week-old mice |  | 247 ± 4 |  |  | 252 ± 6 |  |
| 22-week-old diabetic mice |  | 224 ± 3 |  |  | 237 ± 9 |  |
|  |  |  |  |  |  |  |
| **Contractions to 10 µM NA (N/m)** |  |  |  |  |  |  |
| 12-week-old mice |  | 2.8 ± 0.2 |  |  | 2.7 ± 0.1 |  |
| 34-week-old mice |  | 2.6 ± 0.2 |  |  | 2.7 ± 0.1 |  |
| 22-week-old diabetic mice |  | 3.5 ± 0.3 |  |  | 3.2 ± 0.3 |  |
|  |  |  |  |  |  |  |
|  | **pEC_50_** | **E_max_(%)** | **n** | **pEC_50_** | **E_max_(%)** | **n** |
| **Contractions to 40 mM K^+^** |  |  |  |  |  |  |
| 12-week-old mice | n.d. | 109 ± 5 | 7 | n.d. | 96 ± 6 | 5 |
| 34-week-old mice | n.d. | 100 ± 5 | 6 | n.d. | 104 ± 11 | 6 |
| 22-week-old diabetic mice | n.d. | 104 ± 2 | 7 | n.d. | 92 ± 6 | 4 |
|  |  |  |  |  |  |  |
| **PHE-induced contractions** |  |  |  |  |  |  |
| ***12-week-old mice*** |  |  |  |  |  |  |
| Without inhibitors | 5.8 ± 0.1 | 108 ± 8 | 8 | 5.7 ± 0.1 | 96 ± 3 | 4 |
| INDO | 5.7 ± 0.1 | 104 ± 9 | 8 | 5.6 ± 0.1 | 96 ± 1 | 6 |
| INDO + L-NAME | 5.7 ± 0.1 | 104 ± 8 | 8 | 5.8 ± 0.1 | 96 ± 1 | 6 |
|  |  |  |  |  |  |  |
| ***34-week-old mice*** |  |  |  |  |  |  |
| Without inhibitors | 5.7 ± 0.1 | 102 ± 8 | 5 | 5.8 ± 0.1 | 96 ± 3 | 6 |
| INDO | 5.7 ± 0.1 | 94 ± 6 | 5 | 5.7 ± 0.1 | 96 ± 1 | 7 |
| INDO + L-NAME | 5.7 ± 0.1 | 97 ± 8 | 5 | 5.8 ± 0.1 | 96 ± 3 | 6 |
|  |  |  |  |  |  |  |
| ***22-week-old diabetic mice*** |  |  |  |  |  |  |
| Without inhibitors | 5.8 ± 0.1 | 104 ± 2 | 8 | 5.6 ± 0.1 | 94 ± 6 | 6 |
| INDO | 5.6 ± 0.1 | 104 ± 3 | 8 | 5.5 ± 0.1 | 91 ± 5 | 6 |
| INDO + L-NAME | 5.6 ± 0.1 | 99 ± 3 | 8 | 5.4 ± 0.2 | 89 ± 7 | 6 |

E_max_ values are expressed as % of the maximal response to noradrenaline (NA; 10 µM). All values are shown as mean ± SEM. n.d. = not determined.
