# Supplementary material for: Endothelial Arginine Resynthesis Contributes to the Maintenance of Vasomotor Function in Male Diabetic Mice
Source: PLoS One. 2014 Jul 17;9(7):e102264. doi: 10.1371/journal.pone.0102264 (PMC4102520; doi:10.1371/journal.pone.0102264)
Supplement: Table S3 — Effect of endothelium-specific deletion of ASS on relaxation responses in female mice. Emax expressed as % reduction of the maximal contractile response to 10 µM PHE. All values are shown as mean ± SEM. n.d: not determined. (DOCX) [file pone.0102264.s007.docx]

**Table S3. Effect of endothelium-specific deletion of ASS on relaxation responses in female mice.**

|  |  | **Control** |  |  | **Ass-KO^Tie2^** |  |
| --- | --- | --- | --- | --- | --- | --- |
|  | pEC_50_ | E_max_% | n | pEC_50_ | E_max_% | n |
| **12-wk-old mice** |  |  |  |  |  |  |
| Without inhibitors | 6.7 ± 0.1 | 97 ± 1 | 7 | 6.4 ± 0.7 | 98 ± 2 | 4 |
| INDO | 6.9 ± 0.1 | 93 ± 1 | 8 | 6.8 ± 0.1 | 93 ± 1 | 4 |
| INDO+ L-NAME | 6.6 ± 0.4 | 65 ± 8 | 7 | 6.6 ± 0.2 | 65 ± 6 | 4 |
| Relaxations to SNP | 7.6 ± 0.4 | 98 ± 1 | 5 | 7.4 ± 0.2 | 97 ± 1 | 5 |
| **34-wk–old mice** |  |  |  |  |  |  |
| Without inhibitors | 6.9 ± 0.1 | 85 ± 2 | 7 | 7.1 ± 0.2 | 88 ± 3 | 2 |
| INDO | 6.9 ± 0.1 | 89 ± 3 | 8 | 7.0 | 94 | 1 |
| INDO+ L-NAME | n.d. | 34 ± 5 | 8 | n.d. | 30 ± 4 | 5 |
| Relaxations to SNP | 7.5 ± 0.1 | 96 ± 1 | 4 | 7.4 ± 0.1 | 97 ± 1 | 5 |
| **22-wk-old diabetic mice** |  |  |  |  |  |  |
| Without inhibitors | 6.6 ± 0.2 | 75 ± 19 | 3 | 6.3 ± 0.2 | 49 ± 10 | 3 |
| INDO | 6.5 ± 0.1 | 77 ± 13 | 3 | 6.5 ± 0.2 | 54 ± 10 | 3 |
| INDO+ L-NAME | n.d. | 18 ± 2 | 3 | n.d. | 18 ± 3 | 3 |

E_max_ expressed as % reduction of the maximal contractile response to 10 µM PHE. All values are shown as mean ± SEM. n.d: not determined.
